# A Portable Controllable Compressive Stress Device to Monitor Human Breast Cancer Cell Protrusions at Single-Cell Resolution

**DOI:** 10.3389/fbioe.2022.852318

**Published:** 2022-02-24

**Authors:** Chuan-Feng Yeh, Duane S. Juang, Ya-Wen Chen, Didem Rodoplu, Chia-Hsien Hsu

**Affiliations:** ^1^ Institute of Biomedical Engineering and Nanomedicine, National Health Research Institutes, Miaol, Taiwan; ^2^ Institute of NanoEngineering and MicroSystems, National Tsing Hua University, Hsinchu, Taiwan; ^3^ National Institute of Cancer Research, National Health Research Institutes, Miaoli, Taiwan; ^4^ Ph.D. Program in Tissue Engineering and Regenerative Medicine, National Chung Hsing University, Taichung, Taiwan

**Keywords:** compression force, membrane protrusions, lamellipodia, bleb, single-cell resolution

## Abstract

*In vitro* devices offer more numerous methods than *in vivo* models to investigate how cells respond to pressure stress and quantify those responses. Several *in vitro* devices have been developed to study the cell response to compression force. However, they are unable to observe morphological changes of cells in real-time. There is also a concern about cell damage during the process of harvesting cells from 3D gels. Here we report a device employing transparent, thin gel layers to clamp cells between the interfaces and applied a controllable compression force by stacking multiple layers on the top. In this approach, cells can be monitored for alteration of cellular protrusions, whose diversity has been proven to promote cancer cell dissemination, with single-cell resolution under compression force. Furthermore, p-Rac-1 and rhodamine staining on the device directly to confirm the actin filaments of lamellipodia. The method was able to fulfill real-time live-cell observation at single-cell resolution and can be readily used for versatile cell analysis. MDA-MB-231 and MCF7 breast cancer cells were utilized to demonstrate the utility of the device, and the results showed that the stimuli of compression force induce MDA-MB-231 and MCF7 to form lamellipodia and bleb protrusions, respectively. We envision the device may be used as a tool to explore mechanisms of membrane protrusion transitions and to screen drug candidates for inhibiting cancer cell protrusion plasticity for cancer therapy.

## Introduction

The uncontrollable growth of malignant cells within the tumor generates mechanical compression forces that enhance the capacity of cell motility and invasiveness (Shieh, 2011; Chaudhuri et al., 2018; Kalli et al., 2018). *In vitro* devices offer simple alternative methods that decrease laboriousness, cost, and animal usages compared with *in vivo* models to investigate how cells respond to pressure stress and quantify those responses to gain insight into the compressive forces’ effect on cancer cells (Sachdeva et al., 2021).

There have been attempts to supply compression force using a weight of load on the top of devices applied to cells/cell spheroids, which are on the bottom, to access the cell response. For instance, Tse et al. (2012) seeded cells on a conventional Transwell insert membrane and covered them with a thin film agarose cushion, allowing oxygen and nutrition transmission. This was followed by placing an opaque piston of specific weight on top of the gel to apply a constant force on the cells. Cells formed invasive filopodial protrusions at the leading edge to spread out and disseminate. This novel mechanism has potential to prevent cancer cell migration and invasion. Kim et al. (2017) embedded suspension cells into agarose gel to form a thin film on the bottom of the dish and then applied force to the cells using a cube filled with iron ball bearings on the top. They proved that compression force triggers mechanotransduction to induce vascular endothelial growth factor A (VEGFA) overexpression, thus promoting tumor progression. Kalli et al. (2019) embedded cell spheres into agarose gels and applied a force on the top, following the same method of using the weight of the piston. They discovered compression regulation of brain cancer cell migration *via* the mitogen-activated protein kinase/extracellular signal-regulated kinases (MEK1/Erk1) pathway. The aforementioned setups have provided some useful tools to study how physical compression affects the cellular signal transduction pathways in cancer. However, their applications are limited in end-point analysis because the setup of pressure applied by loads is opaque and real-time observation under microscopy cannot be performed.

To solve this problem, Morikura et al.(Morikura and Miyata, 2019; Morikura and Miyata, 2021) applied stress from the top of the device using a cylindrical hollow weight that allowed observation of cells under compression force and enhanced the invasive elongation of F-actin filaments. This is similar to previous invasive filopodial protrusions of leading edges observed on a 2D surface (Tse et al., 2012). Subsequently, they demonstrated that mechanical intermittent compression can affect the progression rate of malignant melanoma cells in a cycle-period-dependent manner (Morikura and Miyata, 2021). Differing from other static pressure application methods, Novak (Novak et al., 2020) developed a bioreactor device that allows the application of various compression force from 3.9 to 6.5 kPa dynamically to encapsulated cells *via* air pressure, which resulted in cell proliferation, invasion, chemoresistance, and mechanotransduction *via* cell division control protein 42 (CDC42) protein. However, the aforementioned 3D methods share a concern when harvesting the embedded cells from the gel for other analysis, because the collagenase digest technique could damage cells, resulting in the disruption of cell surface molecules (Busser et al., 2014) and cell death (Lee et al., 2015; Claridge et al., 2021).

In this paper, novel device which allows for high-resolution monitoring of the dynamic morphology and migration phenotype of live cells under compression force, and convenient harvesting of the cells from the device is reported. The utility of the device is demonstrated with MDA-MB-231 and MCF7.

## Materials and Methods

### Cell Culture and Transfection

The MDA-MB-231 and MCF7 breast cancer cells, purchased from Bioresource Collection and Research Center (BCRC, Taiwan), were cultured in Roswell Park Memorial Institute (RPMI)-1,640 medium (Biowest) supplemented with 10% fetal bovine serum (FBS) and 1% penicillin-streptomycin at 37°C and 5% CO_2._ Before live-cell monitoring, the cells were transfected with an actin–green fluorescent protein (GFP) reporter using a baculovirus expression vector (CellLight^®^ Actin-GFP BacMan 2.0; Invitrogen) at 70% confluence with a particle per cell ratio of 30 and then incubated at 37°C and 5% CO_2_ for 24 h.

### Preparation of Agarose Gel

As shown in Supplementary Figure S1, the agarose gel layers were fabricated by molding agarose solution with a glass-bottom dish (35 mm µ-Dish; ibidi, Germany) and a glass cover slide. Briefly, low gelling temperature agarose (Type IX-A Ultra-low Gelling Temperature Agarose; Sigma-Aldrich, United States) was dissolved in phosphate-buffered saline (PBS) at 3% concentration by heating in a microwave oven at 600 W for 3 min, followed by mixing the solution with 2X RPMI-1640 cell culture medium supplemented with 20% FBS and 1% penicillin-streptomycin in a 1:1 ratio to form a 1.5% agarose gel solution containing 1X RPMI and 10% FBS, which corresponded to the native osmolality of conventional cell culture media. This ensured the minimal change in concentration and osmolality of the culture media and agarose gel even after a long culture period and also ensured the experimental cells’ culture condition approach that of general cell culture conditions. For the preparation of agarose gel layers, 400 μl of premixed 1.5% agarose solution t was added to a circular glass-bottom dish of 1 mm depth and 21 mm diameter and covered it with a rectangular glass slide (25 mm × 22 mm × 1 mm) (Supplementary Figure S1D). The premixed agarose solution was then allowed to solidify at room temperature, and the glass cover slide was carefully removed to obtain a 21 mm-wide and 1 mm-thick circular gel sheet inside the dish. Finally, the agarose gels, which would be used as an upper (compression) layer, were carefully removed using a spatula and placed on the bottom-layer gels.

### *In Vitro* Compression Device

To apply compression force to cells, the device setup followed a previously published method (Tse et al., 2012). An agarose gel of specified weight applied a constant force to cells, which was seeded on another agarose gel surface, permitting nutrient, and oxygen diffusion. For the experiments, we determined that one gel layer has a weight of 0.38 ± 0.005 g and the area of the gel surface (21 mm diameter) is 346 mm^2^, which translated to a stress of 0.08 mmHg. To increase the degree of compression force, gels were stacked up in multiple layers, which translated to a stress of 0.16 and 0.24 mmHg from the two and three stacked up gels, respectively. Here the compression force is referring to the expected uniform stress from the compressive gels.

### Measurement of Cell Viability and Apoptosis

Cell viability was measured using the Live/Dead cell viability assay kits (Lie/Dead c Kits; Thermo Fisher) after cells were placed under compression force for 24 h. The calcein AM fluorescent indicator was diluted with deionized (DI) water in a ratio of 1:500 (8 μM). The ethidium homodimer fluorescent indicator was diluted with DI water in a ratio of 1:250 (8 μM). Before the experiment, we mixed 2.5 ml each of calcein AM and ethidium homodimer and stocked the mixture in the dark at 4°C. Subsequently, we stained the cells with calcein AM/ethidium homodimer and incubated them for 2 h. After removing the excess dye solution, sterilized PBS was used to wash off the dye remaining in the agarose gels for 1 h at 37°C. An apoptosis assay was performed following the standard protocol of the NucView 488 caspase-3 assay kit (Merck, United States). Finally, we obtained fluorescent images using an inverted microscope (Eclipse Ti, Nikon, Japan) from random locations on the agarose gel.

### Cell Area Measurement

After compressing the cells with an upper layer of agarose gels for 1 h, we placed the glass-bottom dish on an inverted microscope and obtained images from random locations in the central area (5 mm inside the rounded gel edge) using a ×20 objective. The images were processed with the free image-processing software ImageJ (http://rsbweb.nih.gov/ij/) to obtain the single cell area under different compression forces (0, 0.08, 0.16, and 0.24 mmHg).

### Nucleus Surface Area Measurement

After compressing the cells with an upper layer of agarose gels for 4 h, we placed the glass-bottom dish on an inverted microscope and obtained images from random locations in the central area (5 mm inside the rounded gel edge) using a ×40 objective. The cell’s projected nucleus surface area was measured and analyzed with images using NIS-Elements AR 4.1 software (Nikon).

### Time-Lapse Microscopy

Before recording the locomotion and morphology of the cells, we placed the setup on the automated stage of an inverted microscope in an environmental chamber at 37°C and 5% CO_2_ to ensure that the setup is stable. Next, time-lapse images were captured at 1 min intervals for 1 h using an attached charge-coupled device (Retiga 4000DC) and imaging software (Q imaging) with or without epifluorescence, through ×20 objectives. The microscope’s automation was controlled using NIS-Elements AR 4.1 software (Nikon).

### Cell Tracking and Statistical Analysis

The migration trajectories of cells were plotted by the displacements of the centroid of cells determined by the time-lapse images from adjacent time points. We kept the time intervals at 1 min for 1 h. The migration speed was determined by the displacement of the cell centroid as a function of time. The mean speed and standard deviation were calculated with a 95% confidence interval in Excel, and Student’s t-tests were performed. Statistical graphs were generated using Excel, SigmaPlot 12.0 (SPSS), and OriginPro 8.6 (OriginLab).

### Staining of Actin Filaments

Rhodamine phalloidin (Cytoskeleton) was used to stain F-actin in cells after their exposure to compression force for 4 h. Briefly, the cells were fixed in 4% para-formaldehyde for 2 h, washed with PBS for 1 h, and permeabilized with Triton X-100 for 1 h. Subsequently, the cells were stained with 100 nM Rhodamine phalloidin for 2 h, soaked in PBS overnight, and imaged using the inverted microscope equipped with epifluorescence through a standard TRITC filter. As the cells were sandwiched between the upper and bottom layers, this protocol differed in that we used longer incubation and washing times to allow for sufficient diffusion of the reagents compared with the standard protocol for staining on glass slides.

### Immunofluorescence

We performed immunofluorescence staining of cells trapped within the agarose gel setup. The cells were fixed in formaldehyde for 30 min, permeabilized with Triton X-100 for 15 min, then blocked using 2% bovine serum albumin in phosphate-buffered saline with Tween^®^ 20 (PBST) for 2 h. We added primary mouse anti-active Rac1–guanosine-5′-triphosphate (GTP) antibody (NewEast Biosciences) and incubated the cells at 4°C overnight. Alexa-Fluor 488 goat anti-mouse IgG secondary antibody (BioLegend) was added and incubated for 2 h at room temperature, whereas the nucleus was stained using Hoechst for 10 min. All the washing steps were performed using PBS.

## Results

### Design and Operation of the Device

**FIGURE 1:**
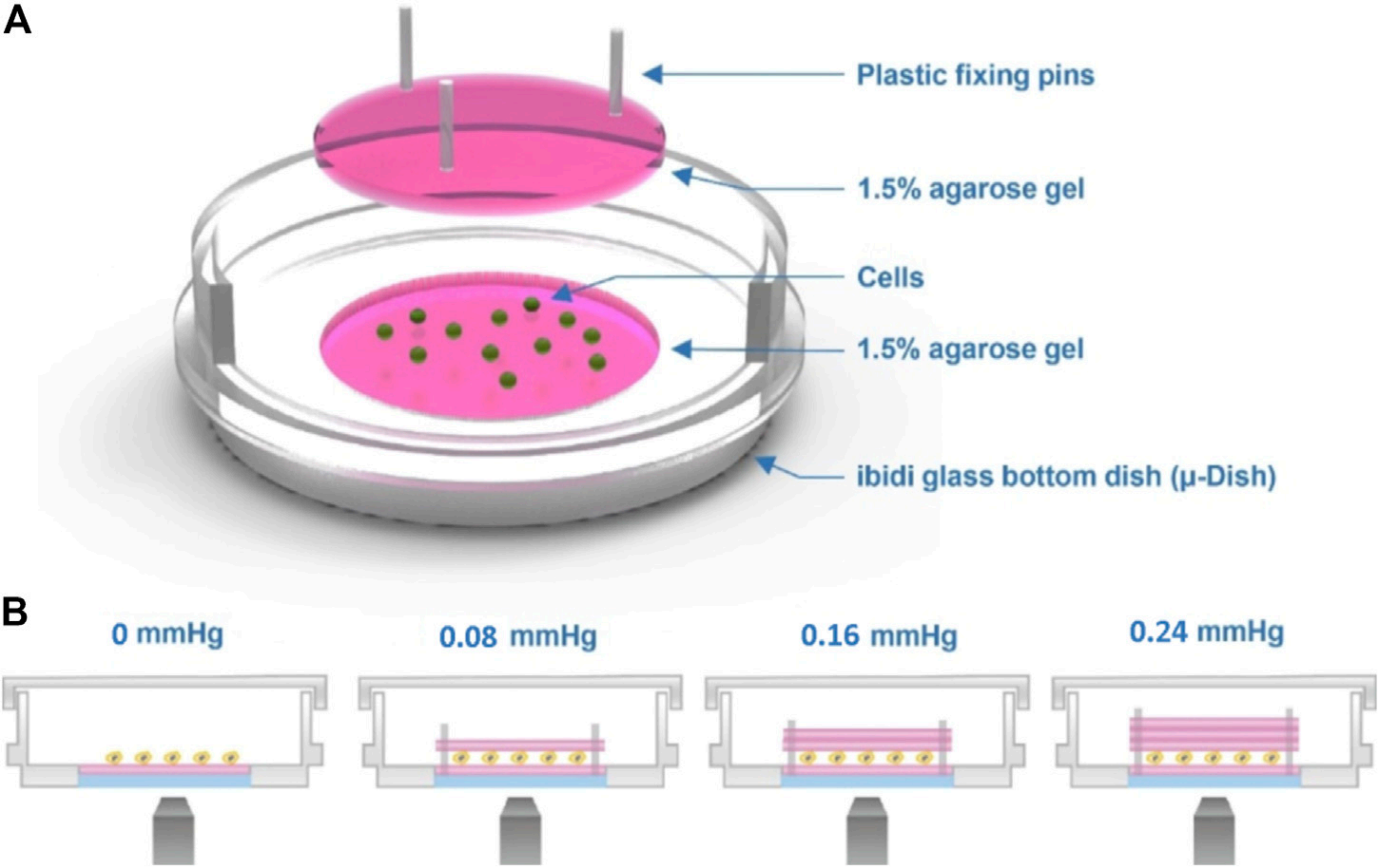
Cell compression experimental setup. **(A)** Schematic of the agarose gel compression method. **(B)** Compression force on the cells is provided by the weight of the top gel. The thickness of the top gel can be varied to obtain different strengths of force, as shown in the image.

The device employs a transparent hydrogel as the load on the top of the cells, which were clamped between the interfaces of agarose gels using the sandwich method. Figure 1 A shows the experimental setup of our agarose gel compression method. In this setup, we controlled the amount of the compression force applied to the cells by varying the thickness of the upper gel layer (Figure 1B). Prior to the experiments, the cells were trypsinized using 1% trypsin–ethylenediaminetetraacetic acid (EDTA), spun down for 3 min at 800 rpm, and resuspended in cell culture medium at a concentration of 5 × 10^5^ cells/ml. The cells were then seeded on the lower gel layer inside the dish, and an upper gel layer was carefully placed on top of the cells (Supplementary Figures 1A, S1F). We used three plastic plugs as pins to fix the position of the lower and upper gel layers and to fix the upper gel layer in place to avoid gel slippage (Supplementary Figure S1G).

### Compression Force Does Not Affect Cell Viability and Apoptosis on the Device

**FIGURE 2:**
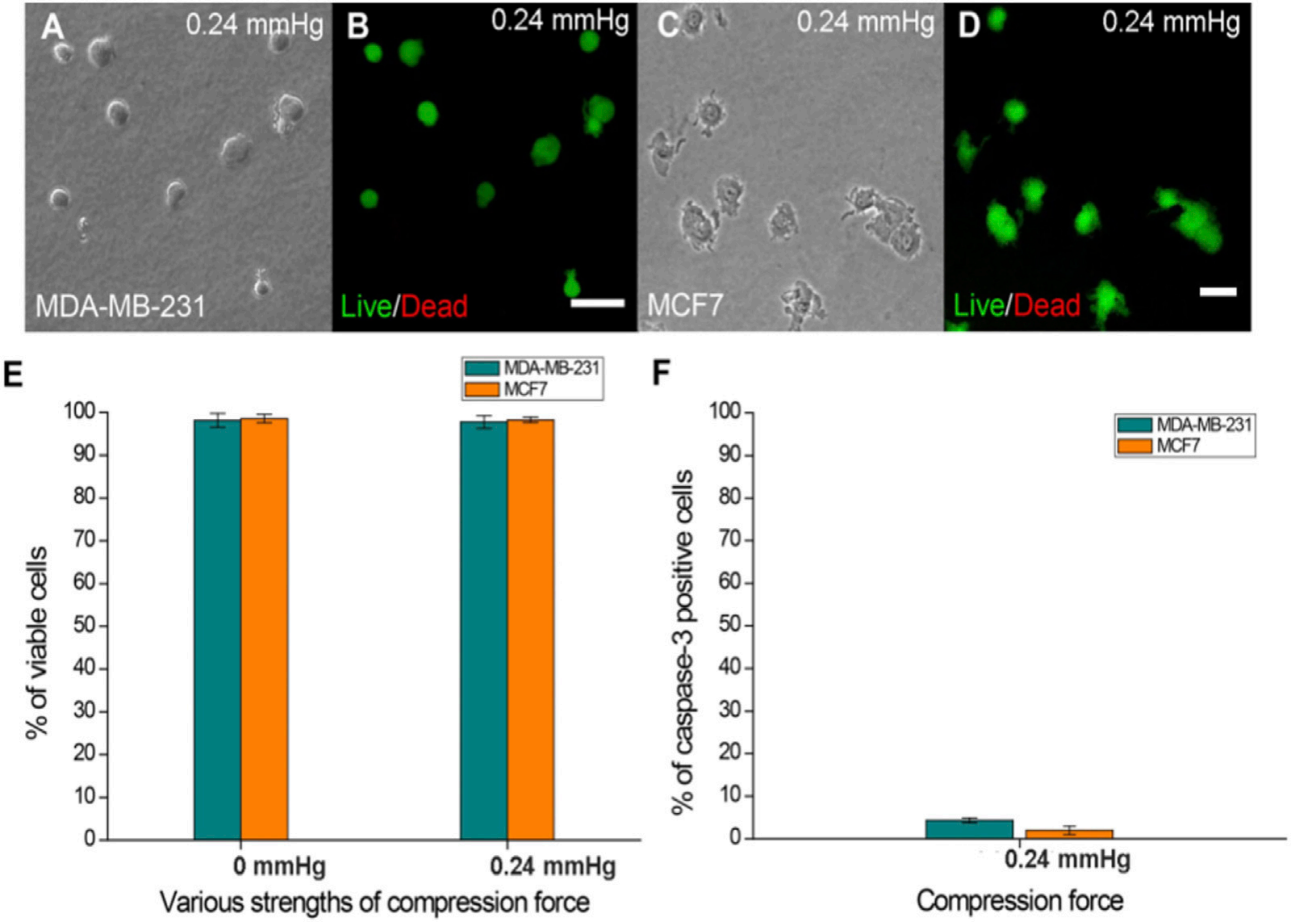
Cell viability of MDA-MB-231 and MCF7 cells after agarose gel compression. **(A)** Bright-field and **(B)** Live/Dead staining of MDA-MB-231 cells under a 0.24 mmHg compression force. **(C)** Bright field and **(D)** Live/Dead staining of MCF7 cells (green fluorescence) was observed under a 0.24 mmHg compression force. Scale bar = 100 μm. **(E)** Both cell lines showed approximately 97% viability on the agarose gel surface without compressing gels. The cell viability of MDA-MB-231 and MCF7 cells (both approximately 97%) was not affected by 0.24 mmHg compression force. **(F)** Caspase-3-positive MDA-MB-231 and MCF7 cells were all less than 5% under 0.24 mmHg compression force.

To understand whether the applied compression force could cause cell damage which can affect the response of cells to compressive stress, cell viability and apoptosis assays were performed on cells treated with the largest compression force (0.24 mmHg) for 24 h. The results showed no significant difference in viability between compressed and uncompressed cells in the largest force used (Figure 2E), and the percentage of caspase-3-positive cells was less than 5% in both MDA-MB-231 and MCF7 cells (Figure 2F). The data showed that cells have good viability under compressive force after 24 h. The experiments were implemented for 24 h to ensure that cells present optimal conditions.

### Compression Force Induces MDA-MB-231 Breast Cancer Cell Morphology Change and Enlarges Cell Area and Lamellipodia Protrusions in a Force- and Time-Dependent Manner

**FIGURE 3:**
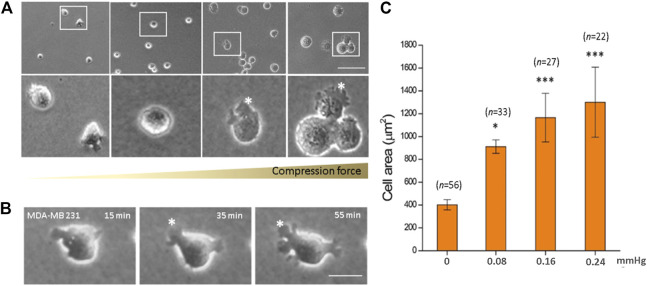
Compression force enlarged the cell area of MDA-MB-231 cells on a low-adhesive surface. **(A)** The cell area of MDA-MB-231 cells significantly enlarged with increasing compression force from 0 mmHg (left) to 0.08, 0.16, and 0.24 mmHg (right) after culturing for 1 h. **(B)** An MDA-MB-231 cell showing enlarged membrane protrusions which increased with time under 0.24 mmHg compression force. **(C)** Quantitative analysis of the individual cell area after 1 h treatment with various compressive stress values. The cell area significantly increased from 401.67 ± 44.59 μm^2^ (0 mmHg) to 1,300.06 ± 307.37 μm^2^ (0.24 mmHg). Scale bar = 100 μm *p < 0.05; ***p < 0.005. Student’s t-test, two independent experiments.

We observed a significant increase in the cell area in response to increased compression force. This was because as the cells flattened, they spread out more and presented lamellipodia protrusions in the cell front. Quantitative results showed that on increasing the compression force, the MDA-MB-231 cell area significantly increased on a low-adhesive surface (Figure 3A). The cell area significantly increased from 401.67 ± 44.59 μm^2^ (without a gel covering, 0 mmHg) to 1,300.06 ± 307.37 μm^2^ (covered by three gels; 0.24 mmHg) (Figure 3C, *p* < 0.001). This shows that the compression force could induce MDA-MB-231 cell morphology changes and lamellipodia protrusions in a time-dependent manner. Cell protrusions were observed after 15 min under compression, which gradually enlarged until 55 min, when they were the largest (Figure 3B).

### Compression Force Regulates the Ratio of Blebs or Lamellipodia Protrusion on a Low-Adhesive Surface

**FIGURE 4:**
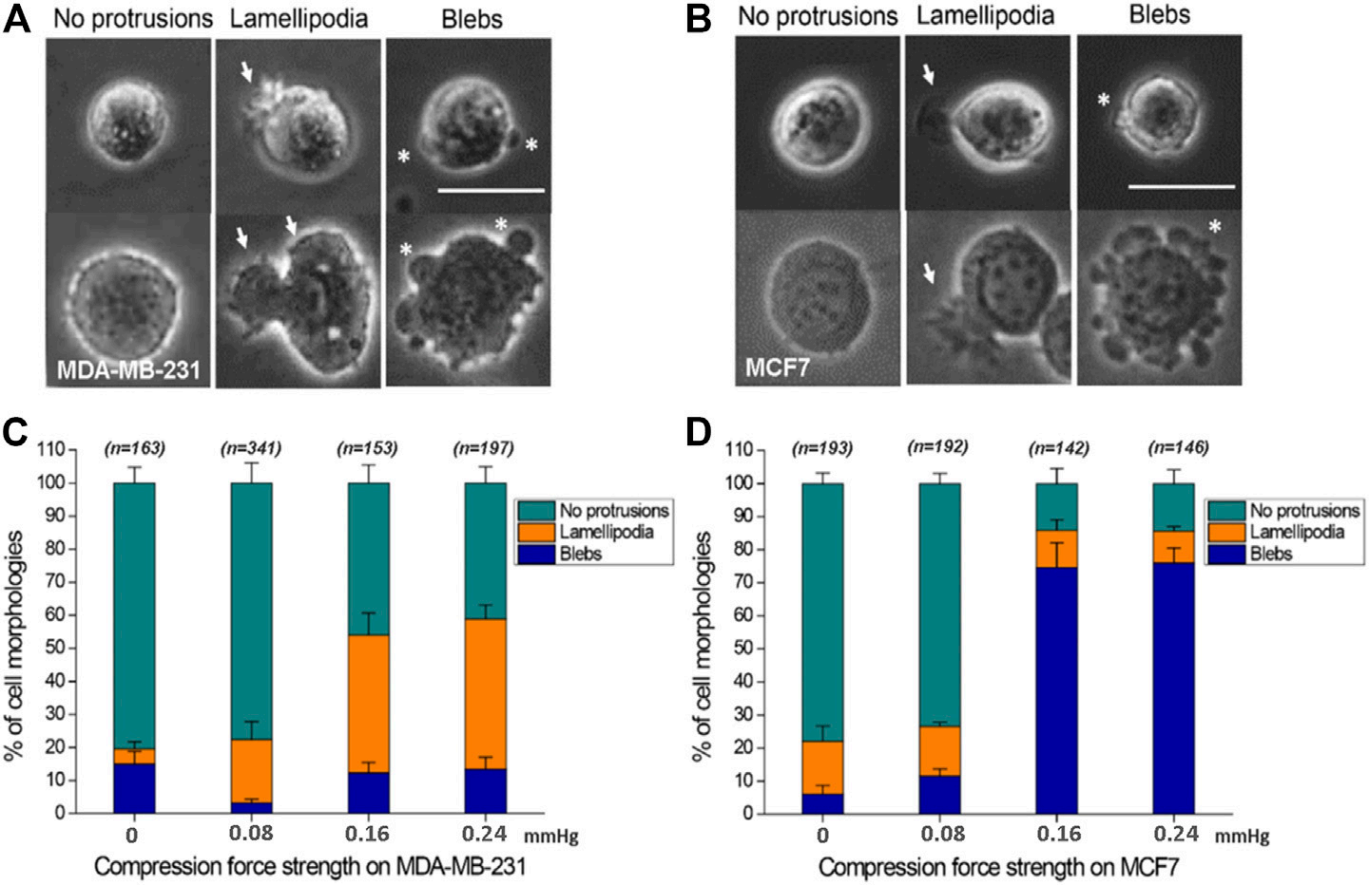
Compression force regulates cell protrusions to transition from no protrusions to lamellipodia or blebs in MDA-MB-231 and MCF7 cells on a low-adhesive surface. **(A, B)** Representative images of the three cell membrane protrusions, including (1) no protrusions, (2) lamellipodia, and (3) blebs, in **(A)** MDA-MB-231 and **(B)** MCF7 cells on a low-adhesive surface with (below) or without (above) compression force. The white arrows indicate lamellipodia, and the stars show blebby protrusions on the cell membrane. Scale bar = 30 μm. **(C)** MDA-MB-231 cells displayed a transition from no protrusions to lamellipodia. The ratio of lamellipodia is related to the strength of compression force. **(D)** Compression force regulated the membrane protrusion transition of MCF7 cells from no protrusions to blebs on a low-adhesive surface. The ratio of blebs is positively related to the strength of compression force. Quantitative analysis of cell protrusion type conditions was performed by manually identifying each individual cell’s protrusion types from the whole images obtained under the 0 mmHg, 0.08 mmHg, 0.16 mmHg and 0.24 mmHg compression force condition, and the total counted cell number for each condition is shown in Figure C and D.

Here, the effect of mechanical force on cells was studied in the absence of adhesion between cell and substrate. To the best of our knowledge, the effect of compression force on regulating cancer cell protrusion and migration in the absence of cell–matrix adhesion is unclear. Application of compression force to MDA-MB-231 and MCF7 cells using the compression assay induced a dramatic switch in the cellular membrane from no protrusions to lamellipodia (MDA-MB-231) or blebs (MCF7) on the low-adhesive agarose gel surface (Figures 4A–D). Similar results were observed with cells stained with Rhodamine phalloidin (Supplementary Figure S2) or transfected with actin-GFP using a baculovirus vector to visualize actin filaments. The transition of cell membranes from no protrusions to lamellipodia was mostly observed in MDA-MB-231 cells (Supplementary Movie S1), a high-invasive (Hooshmand et al., 2013; Aumsuwan et al., 2016; Kwiatkowska et al., 2016; Dong et al., 2020; Samandari et al., 2021) and high-migratory-ability breast cancer cell line, where in the ratio of lamellipodia increased from 4.57% (0 mmHg) to 45.43% (0.24 mmHg) (Figure 4C). The lamellipodia protrusions of many of the cells expanded in a dynamic, time-dependent manner (Figure 3B). Large lamellipodia formations have been observed in high-invasive cells, such as glioma cells (Ryu et al., 2012), osteoblast-like cells, hepatoma cells, and MDA-MB-231 cells on an adhesive surface without compression force. However, we found that the low-invasive MCF7 (Hooshmand et al., 2013; Aumsuwan et al., 2016; Kwiatkowska et al., 2016; Dong et al., 2020; Samandari et al., 2021) cells showed a major increase in bleb protrusions from 6.12% (0 mmHg) to 76.00% (0.24 mmHg) (Figure 4D) instead of lamellipodia extensions. The plasticity of cell membrane protrusions as an adaption to the cellular microenvironment plays a critical role during cell migration. Thus, these results demonstrated that a typically adhesive cell membrane protrusion phenotype (lamellipodia) can be induced by mechanical compression force in high-invasive cells (MBA-MD-231) (Hooshmand et al., 2013; Aumsuwan et al., 2016; Kwiatkowska et al., 2016; Dong et al., 2020; Samandari et al., 2021) but not in low-invasive cells (MCF7) (Hooshmand et al., 2013; Aumsuwan et al., 2016; Kwiatkowska et al., 2016; Dong et al., 2020; Samandari et al., 2021) regardless of the absence of cell–substrate adhesion.

### Breast Cancer Cells Display High Motility on a Low-Adhesive Surface

**FIGURE 5:**
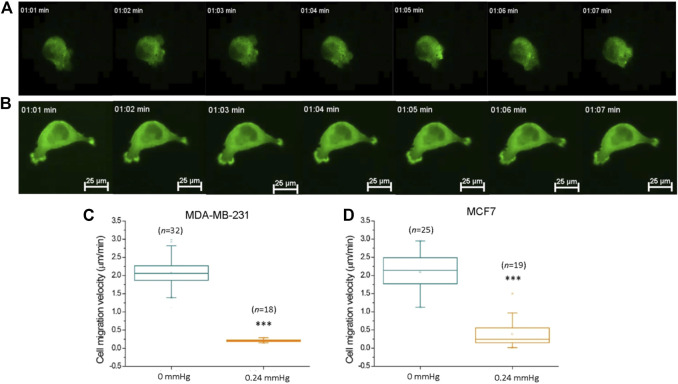
Compression force suppressed MDA-MB-231 cancer cell motility on a low-adhesive surface. **(A)** Representative time-lapse images of an actin-GFP-transfected MDA-MB-231 breast cancer cell displaying locomotion on a low-adhesive agarose gel surface without compression force. **(B)** The motility of MDA-MB-231 cells decreased with 0.24 mmHg compression force on the agarose gel surface during a 60-min durations. Compression force significantly suppressed **(C)** MDA-MB-231 and **(D)** MCF7 cell motility on a low-adhesive surface. The cell’s velocity was calculated and analyzed with time-lapse images using MetaMorph software. Scale bar = 25 μm ***p < 0.005. Student’s t-test, three independent experiments. GFP, green fluorescent protein.

MDA-MB-231 and MCF7 cells were seeded on top of the agarose gel surface without covering them with an upper gel layer. Interestingly, these cells showed locomotion on the 2D agarose surface without cell–substrate attachment (Figure 5A and Supplementary Movie S2). To confirm that the cell movement is autonomous instead of being caused by fluid convection, the cells were co-seeded with polystyrene microbeads whose size and mass density were close to those of the cells (1.05 g/cm^3^, 10 μm diameters, Sigma-Aldrich). The microbeads remained stationary during the experiment, indicating that cell movement is indeed autonomous (Supplementary Movie S3). We analyzed the obtained time-lapse micrographs of the cells and found that the average velocity of the cells was 1.76 ± 1.29 μm/min, which is faster than that previously reported in both 3D matrigel and a 2D culture dish (Torka et al., 2006). Interestingly, in contrast to the previous belief that cells lack the ability to migrate on non-adhesive 2D surfaces (Lammermann and Sixt, 2009), this result showed that cancer cells are motile on the low-adhesive gel surface, with majorly blebby protrusion movement.

### Compression Force Suppresses Cell Motility on a Low-Adhesive Surface

Previous studies have demonstrated that compression force enhances the extension of long filopodia (actin protrusions at the tips of lamellipodia) and promotes cancer cell migration through stronger focal adhesions (Tse et al., 2012). In addition, migratory cell speed can be mechanically controlled by the shape of the cellular mechanisms involving the cytoskeleton or adhesive contacts (Seetharaman and Etienne-Manneville, 2020; Shellard and Mayor, 2020). To investigate whether cell motility is promoted or demoted by compression force on a low-adhesive surface, we subjected MDA-MB-231 cells in our compression experiment setup to 0.24 mmHg of compression force (Figure 5B) and recorded the cells’ locations and morphologies using time-lapse microscopy at 1 min intervals for 60 min. To confirm that the cell movement is not caused by lateral drift of the stacked gel, we analyzed the migration trajectories of individual cells from the same field view, and found that the individual cells were moving toward different directions, indicating that the cell movement is autonomous (Supplementary Figure S3, Supplementary Movie S4). Compression force significantly reduced the motility of MDA-MB-231 and MCF7 cells on a low-adhesive surface (Figures 5C,D). Our finding suggests that compression force and the confined space surrounding the cells have the combined effect of impeding cell movement in a low-adhesive environment. However, a systemic investigation of low-magnitude-force distribution during migration on a low-adhesive surface is required in order to clarify the locomotion model in the future (Paluch et al., 2016).

### Compression-Force-Induced Lamellipodia Formation on the Low-Adhesive Surface Require Phosphorylation of Rac-1

**FIGURE 6:**
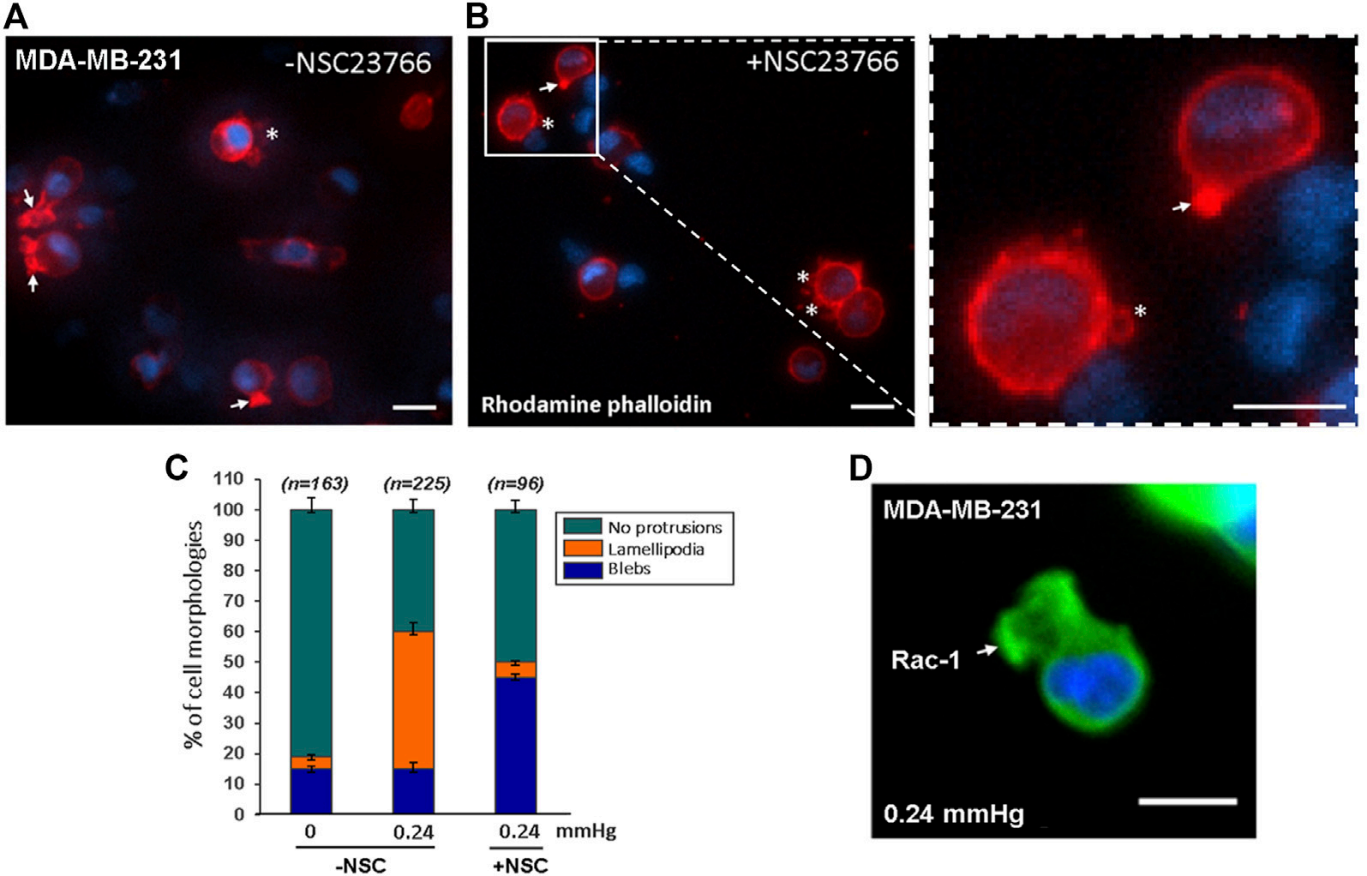
Compression force induced lamellipodia protrusions of MDA-MB-231 cells on low-adhesive surface were partially suppressed with Rac-1 inhibitor. **(A)** 0.24 mmHg compression force induced lamellipodia protrusions formation in MDA-MD-231 cells without Rac-1 inhibitor. **(B)** Rac-1 inhibitor partially suppressed compression force induced lamellipodia protrusions in MDA-MB-231 cells. The white arrows indicate lamellipodia, and the stars show blebby protrusions on the cell membrane. Scale bar = 20 μm. **(C)** Quantitative analysis of the ratio of cell protrusion types in different conditions (-compression force/-Rac-1 inhibitor; +compression force/-Rac-1 inhibitor; +compression force/+inhibitor) from images obtained in two independent experiments. **(D)** Immunostaining of phosphorylated Rac1 protein in cells under 0.24 mmHg compression force revealed that Rac1 is expressed near the edge of the cellular lamellipodia protrusions. Scale bar = 20 μm.

In general, cell-substrate adhesion is formed by the binding of specific transmembrane signaling molecules such as integrin to extracellular matrix (ECM), which initiates intracellular network signaling leading to actin-polymerization driven lamellipodia formation and cell migration on the ECM substrate (Mgrditchian et al., 2021; Thompson et al., 2021). Under cell-substrate adhesion condition, the lamellipodia formation at the cell’s front edge is stimulated by Rac-1 activation (Mehidi et al., 2019; Schaks et al., 2021). However, since lamellipodia formation in low cell-substrate adhesion conditions were not observed in previous studies, it was not clear whether the compression force-induced lamellipodia protrusions observed in our low-adhesive system also requires Rac-1 activation. To answer this question, we compared compression force induced-lamellipodia formation of Rac-1 inhibitor NSC23766 treated MDA-MB-231 cells to that of untreated MDA-MB-231 cells on low adhesive surface. Our results showed that unlike the untreated cells, in which compression force robustly increases lamellipodia protrusions from 4% (non-compressed) to 45% (compressed), the NSC23766treated cells did not show increased lamellipodia formation from compression force under the low adhesive substrate condition (Figure 6). This data showed that the compression-force-induced lamellipodia protrusions under low adhesive substrate were at least partially stimulated by Rac-1 activation. We also confirmed the Rac-1 protein’s *in situ* expression at the front edge of the lamellipodia in the low adhesive surface under compression force (Figure 6D). Together, these results show that Rac-1 is also involved in lamellipodia protrusion formation induced by compression force in low adhesive surfaces.

## Discussion

Previous studies have established several *in vitro* devices to investigate the influence of compression forces on cancer cells (Table 1). The gel-embedded-cell methods (Kim et al., 2017; Kalli et al., 2019; Morikura and Miyata, 2019; Novak et al., 2020; Morikura and Miyata, 2021) provide 3D conditions that better mimic *in vivo* environments than 2D methods, but are limited by the requirement of using collagenase to harvest gel-embedded-cells. To address this limitation, a sandwich method that allowed the cells to be easily harvested after the cell compression experiment was developed (Meyer et al., 2012). This sandwich method provides straightforward cell harvesting functionality needed for various molecular quantitative analysis. However, due to the need of using an opaque piston as the load, does not allow real-time observation of the cells during the compression period (Tse et al., 2012). Different from the sandwich method, our device uses stacked semi-transparent agarose gel to apply compression force, so besides easy cell harvesting it can also allow for real-time observation of the dynamic morphological change and cell migration behaviour of the cells during compression experiments. Increasing evidence indicates that compression force induces cancer cells to alter their morphology and protein expressions to allow them to retain their migration capability in different environmental challenges (Fort et al., 2018; Holle et al., 2019; Alexandrova et al., 2020). Although the plasticity of cellular membrane protrusions is known to be involved in cancer cell migration and metastasis, how compression force influences these protrusions on a low-cell-adhesion surface is unclear. Our device provides a new tool to study cancer cell protrusion changes under compression force in a low cell-substrate adhesion condition may help gain new insights into cancer metastasis and potentially lead to new treatment strategies (Broders-Bondon et al., 2018; Fort et al., 2018; Alexandrova et al., 2020). In addition, our device may also be used for compression experiments on adhesive surfaces with a small modification; for example, seeding the cells on the glass substrate of the commercial 35 mm µ-Dishes instead of on the gel substrate. By using different gel materials (e.g., polyacrylamide gel) or agarose gels of different agarose concentrations, our device could also be used to study the effect of compression forces under different substrate elasticity conditions.

**TABLE 1:** Comparison of *in vitro* compression devices.

Methods of force application	Weight of the piston on the top Tse et al. (2012)	Weight of the cube filled with iron ball bearings Kim et al. (2017)	Weight of the piston on the top Kalli et al. (2019)	Cylindrical weight on the top Morikura and Miyata (2019)	Cylindrical weight on the top Morikura and Miyata (2021)	Air pressure applied via a bioreactor device Novak et al. (2020)	Weight of gels on the top [this work]
Compression force	5.8 mmHg	5.8 mmHg (∼0.773 kPa)	4 mmHg	0.77 kPa	0.77 kPa	3.9–6.5 kPa dynamically	0.08–0.24 mmHg
Compression force applied duration	16 h	24 h	16 h	8 and 32 h	a cycle of 4 h on/4 h off	24 and 72 h	1, 2, 4, and 24 h
Cell types	Breast cancer cells	Breast cancer cells	Brain cancer cells	Melanoma cells	Melanoma cells	Ovarian cancer cells	Breast cancer cells
Cell culture condition	Cells sandwiched between gels	Cells embedded in agarose	Spheroids embedded in agarose	Cells embedded in collagen	Cells embedded in collagen	Cells embedded in agarose/collagen	Cells sandwiched between gels
Real-time observation	No	No	No	Yes	Yes	No	Yes
Single cell resolution	No	No	No	No mentioned	No mentioned	No	Yes
Hydrogel digest for cell harvest	Not required	Required	Required	Required	Required	Required	Not required

The range of the typical compression forces that cells experience within a tumor remains an outstanding question due to the lack of direct measurements *in vivo* (Pratt et al., 2020)*.* Hence, previous studies (Tse et al., 2012; Kim et al., 2017; Kalli et al., 2019; Morikura and Miyata, 2019; Novak et al., 2020; Morikura and Miyata, 2021) utilized the maximum forces that did not induce cell apoptosis as the upper limit of the compression forces (ranging from ∼0.77 to 6.5 kPa) for their experiments. In our case, we observed that cancer cell protrusion, migration and nucleus morphology (Supplementary Figure S4) could be altered even under a relatively smaller compression range (∼0.011–0.032 kPa). In additions, we performed confocal imaging and analysis to obtain the 3D rendering of the gel-cell-gel interface. The results showed that the gels’ topographies are well conformed to that of the cell, indicating there is no gap between the cell and the gel surfaces, and also revealed that the cell is flattened by the compression stress, and the larger the compression force the flatter the cell’s morphology becomes (Supplementary Figure S5).

When cells are free from an external compression force, two types of single cell migration are typically observed, referring to the mesenchymal and amoeboid migration modes. The mesenchymal mode is usually observed from cells on 2D adherent surfaces and is characterized by an elongated spindle shape morphology with actin-polymerization driven lamellipodia protrusions at the cell front *via* Rac-1 and CDC 42 and the retraction of the cell rear regulated by the release of adhesive contacts from extracellular matrix proteins (Sackmann, 2015; Lawson and Ridley, 2018), whereas the amoeboid mode is observed in low-adhesion 3D conditions and is characterized by a circular morphology with actomyosin contractile membrane blebbing (Agarwal and Zaidel-Bar, 2019; Guzman et al., 2020). Previous studies have shown that compression force could induce filopodial protrusions (Tse et al., 2012) (i.e., enhance lamellipodia protrusions) of cells on 2D adherent surfaces, and induce lamellipodia protrusions (Morikura and Miyata, 2019) of cells in 3D low-adhesion conditions. However, how compression force affects cells on 2D low-adhesive surfaces remained unclear. Our results show that in 2D low-adhesion conditions, compression force induces a transition from no protrusions to lamellipodia for MDA-MB-231 and no protrusions to blebs for MCF7. These protrusion changes were not observed in both 2D adherent and 3D low-adhesion conditions. We also found that force-induced lamellipodia protrusions in MDA-MB-231 can be suppressed by Rac-1 inhibitor and transition to bleb protrusions.

Another interesting finding, we observed is that in the compressed condition of our setup, the cells retain their capacity to migrate in between the low-adhesion sandwich surfaces at decreased velocity. This decreased migration speed contrast with the findings of a previous study which showed an increased cell migration speed when cells were in a compressed 2D adhesive surface condition (Tse et al., 2012). A possible explanation to these opposite results is that, for a cell sandwiched between two gels to move forward, the driving force, which is generated from the friction force between the cell’s membrane and its surrounding gel surfaces needs to be larger than the resisting force which is caused by the cell needing to open up a gap between the two gel surfaces in front of the cell for the cell to pass through. The increase of compression force increases both the driving force and resisting force. However, the driving force and resisting force are increased differently in adhesion and low-adhesion conditions; in adhesive conditions, the driving force is increased more than the resisting force, resulting in a gain of the migration speed of the cell whereas, in the low adhesion condition, the increased driving force is not enough to compensate for the increased resisting force resulting in a lowered migration speed. In summary, the observed cell migration in between low-adhesion sandwich surfaces depends on the balance the friction and resisting forces in the setup.

## Conclusion

We demonstrated a convenient method for easy setup to monitor cancer cells’ response under compression forces in real-time at single-cell resolution by time-lapse imaging in general laboratories. The experimental cells between gels in the device could be conveniently and easily harvested without needing a gel digest process for subsequent gene or protein expression assays. However, the method may not be suitable for high compression force experiment owing to the need for stacking several gel layers which might not fit in a regular dish. Our results show that the pro-posed device offers an alternative tool to study the effect of compression force and chemical factors on cancer cells to help understand the correlation between cellular protrusion change and cancer metastasis, and has the potential to be utilized in screening candidate drugs that inhibit the protrusion plasticity of cancer cells to suppress cancer metastasis.

## Data Availability

The original contributions presented in the study are included in the article/Supplementary Material, further inquiries can be directed to the corresponding author.
