# Supplementary figures and images for: A Portable Controllable Compressive Stress Device to Monitor Human Breast Cancer Cell Protrusions at Single-Cell Resolution
Source: Front Bioeng Biotechnol. 2022 Feb 24;10:852318. doi: 10.3389/fbioe.2022.852318 (PMC8907972; doi:10.3389/fbioe.2022.852318)

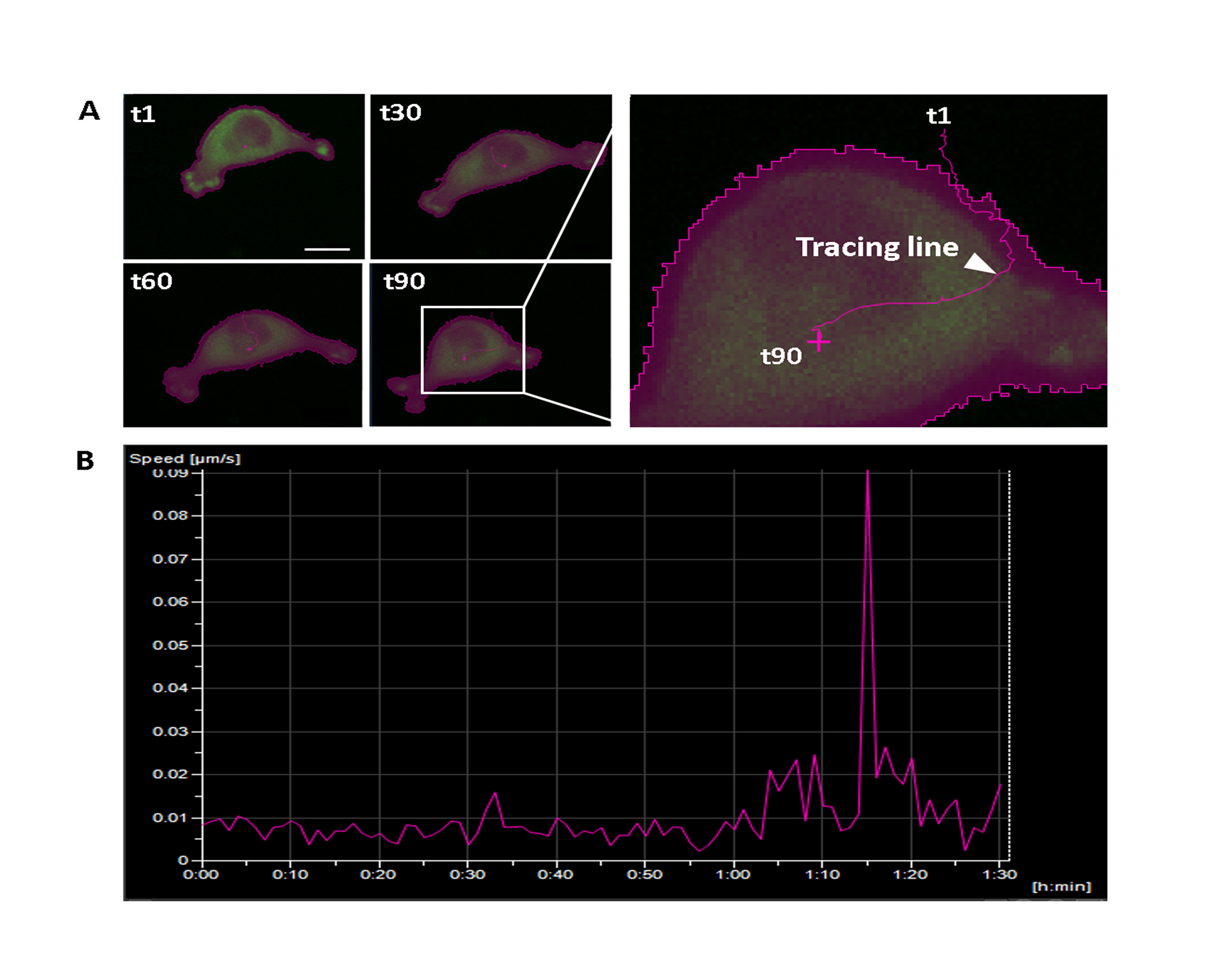

Supplement: Supplementary file 3 [file Image3.TIF]

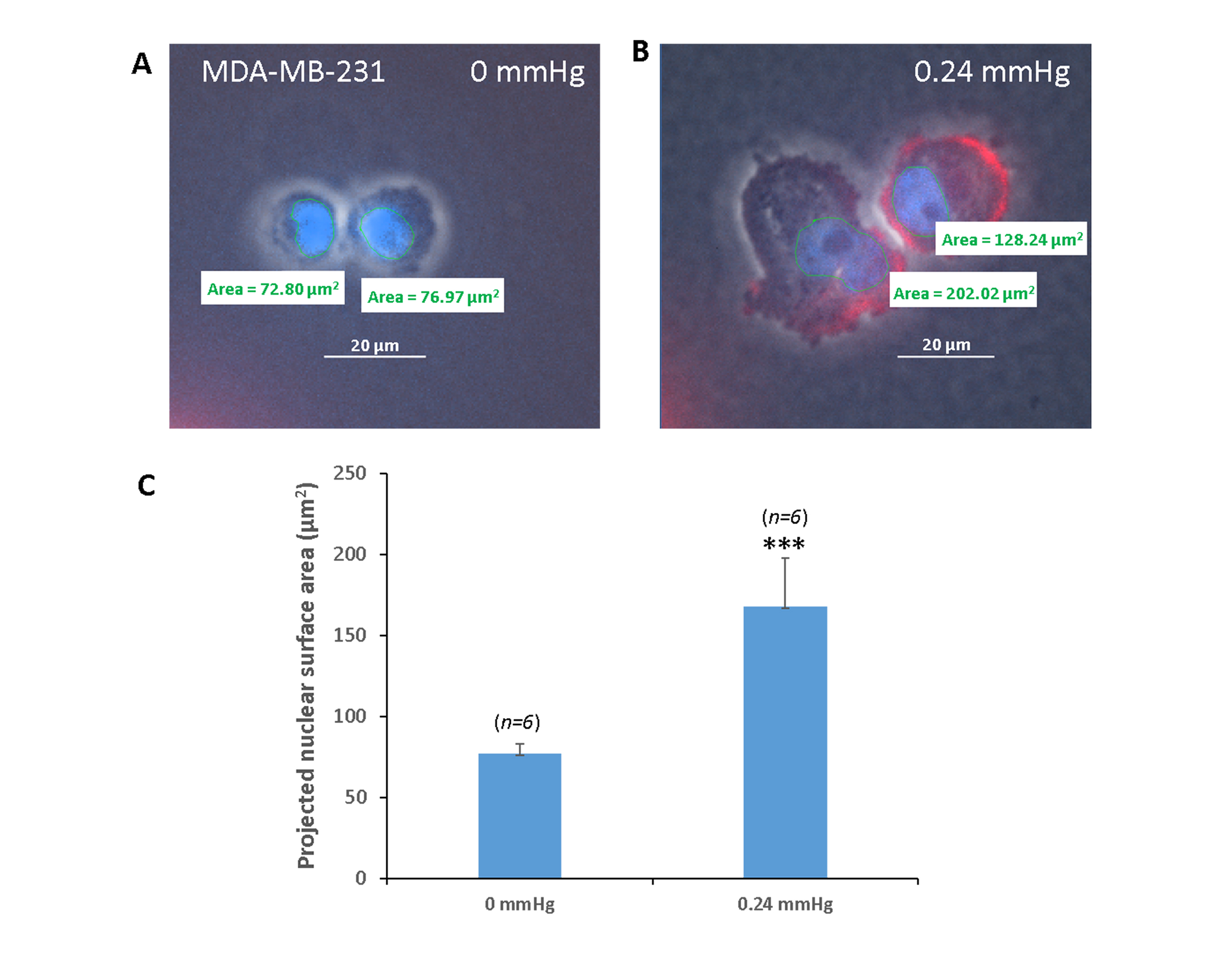

Supplement: Supplementary file 4 [file Image4.TIF]

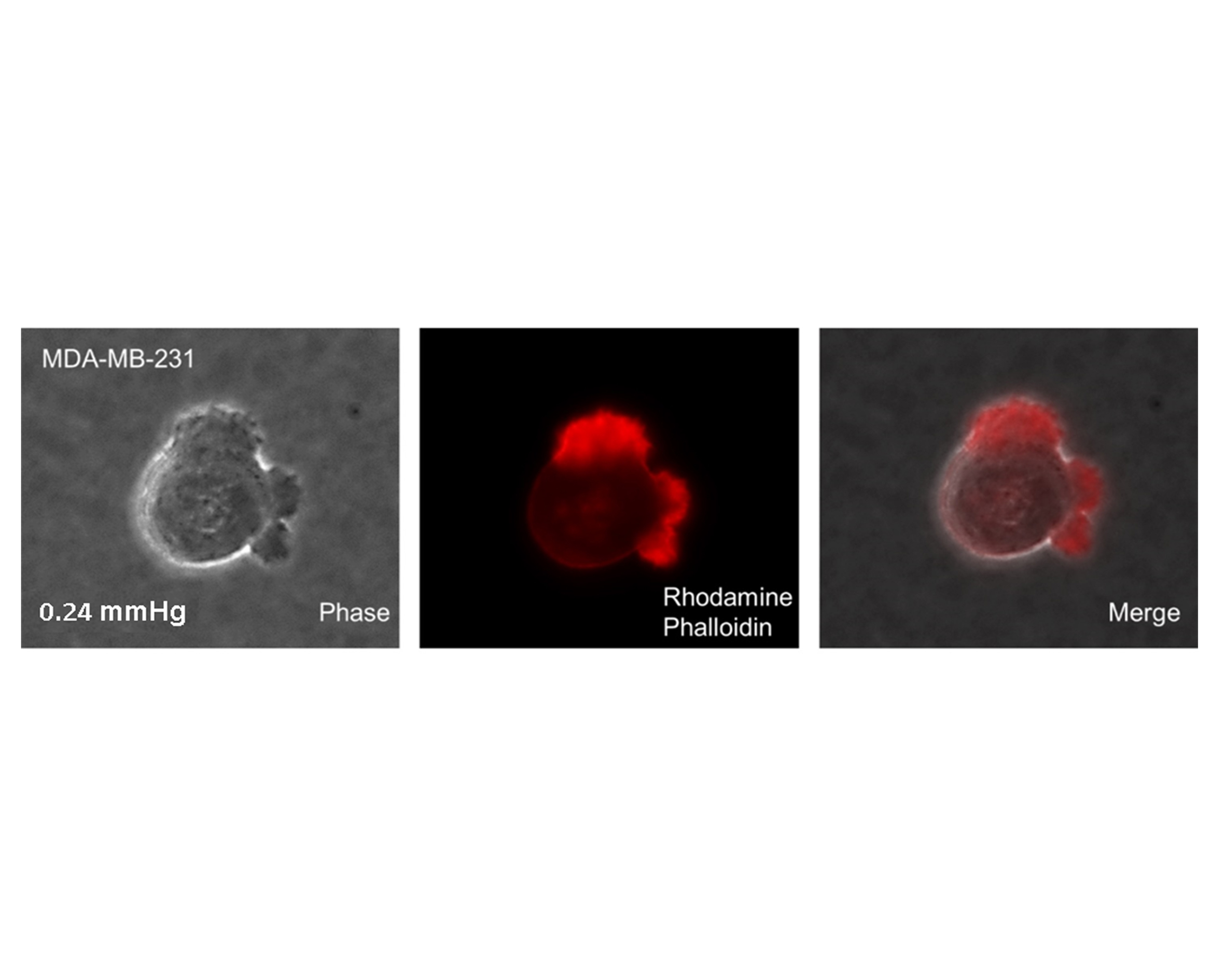

Supplement: Supplementary file 5 [file Image2.TIF]

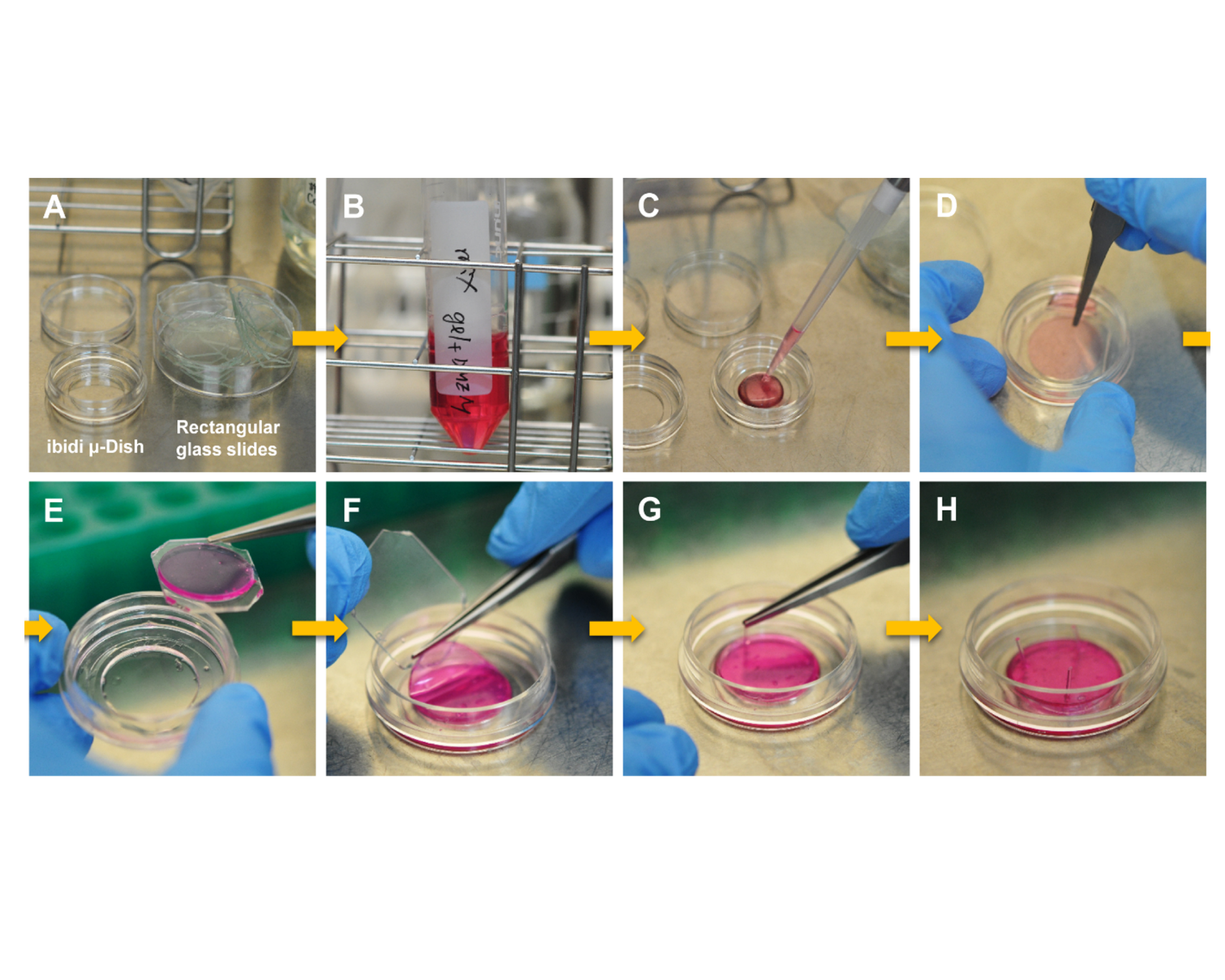

Supplement: Supplementary file 6 [file Image1.TIF]

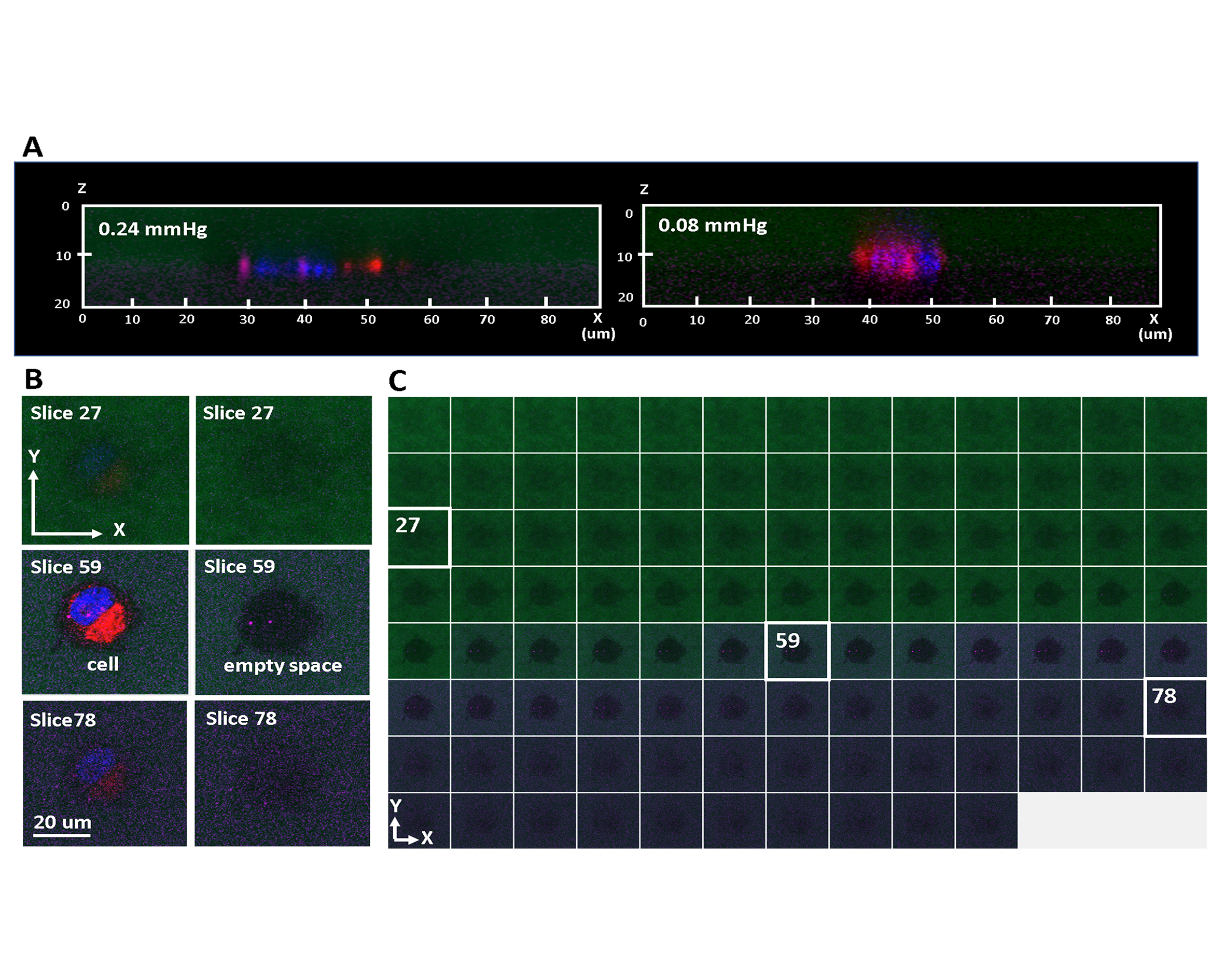

Supplement: Supplementary file 8 [file Image5.TIF]
