# Supplementary material for: A Portable Controllable Compressive Stress Device to Monitor Human Breast Cancer Cell Protrusions at Single-Cell Resolution
Source: Front Bioeng Biotechnol. 2022 Feb 24;10:852318. doi: 10.3389/fbioe.2022.852318 (PMC8907972; doi:10.3389/fbioe.2022.852318)
Supplement: Supplementary file 10 [file DataSheet1.docx]

Supplementary Material

A Portable Controllable Compressive Stress Device to Monitor Cancer Cell Protrusions at Single-Cell Resolution

Chuan-Feng Yeh, Duane S. Juang, Ya-Wen Chen, Didem Rodoplu and Chia-Hsien Hsu

## Supplementary Figures


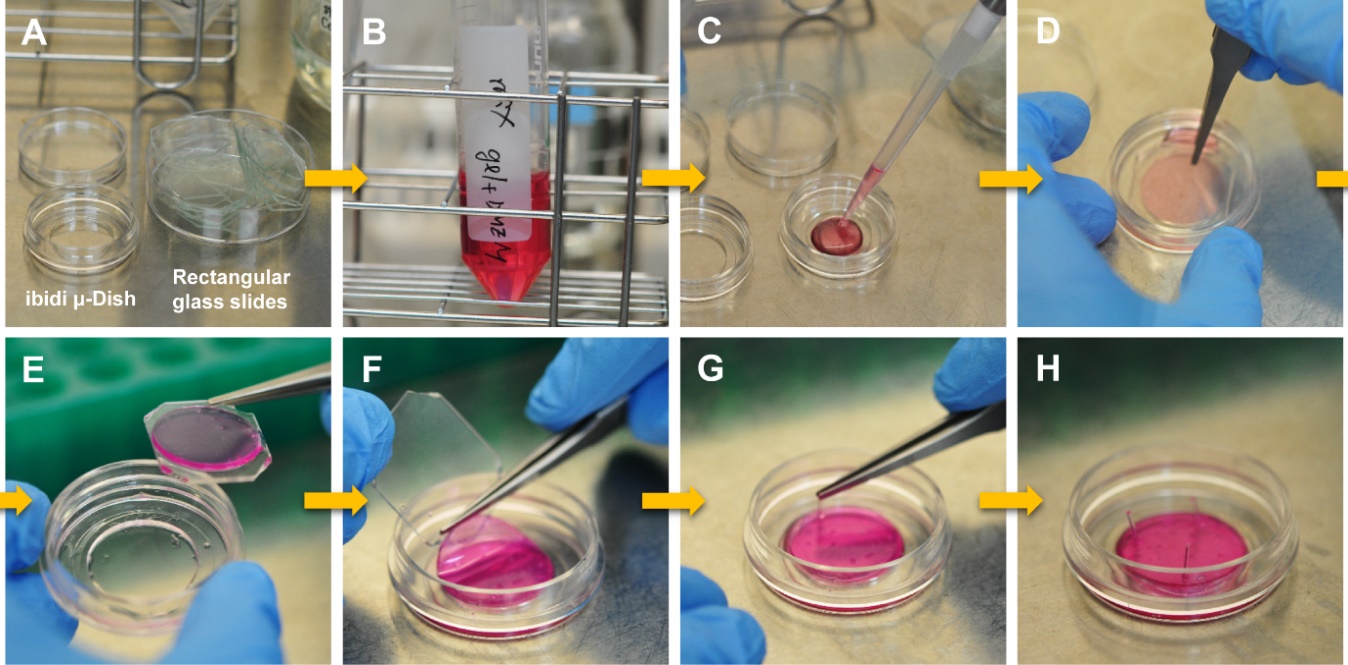


**Supplementary Figure 1.** Operation workflow of agarose gel casting and compression assay setup. (A) Before the experiments, an ibidi μ-Dish and rectangular cover slides are prepared for agarose gel casting. (B) 3% agarose gel solution and 2× culture medium was mixed at a volume ratio of 1:1 to produce a 1.5% agarose gel solution containing 1× culture medium. (C) 400 μL of the premixed 1.5% agarose solution is added to the well of μ-Dishes. (D) After adding the gel solution, a rectangular glass slide is placed on the top of the gel solution to squeeze out the excess solution and air bubbles to form an agarose gel sheet 20 mm in diameter and 1 mm in thickness. The gelled agarose gel sheet is left in the μ-Dish as a bottom gel or can be (E) removed from the μ-Dish to be used as a top gel. (F) After cell seeding, an upper agarose gel is placed on the top of the bottom agarose gel, and aligned by the gels' edges. (G) Plastic pins are used to fix the upper gel and bottom gels, preventing the top gel from sliding sideways while handling the dish. (H) Finally, the dish can be covered (lid not shown in the picture) to complete the gel compression assay setup for cell experiments with an environmental chamber-equipped microscope.


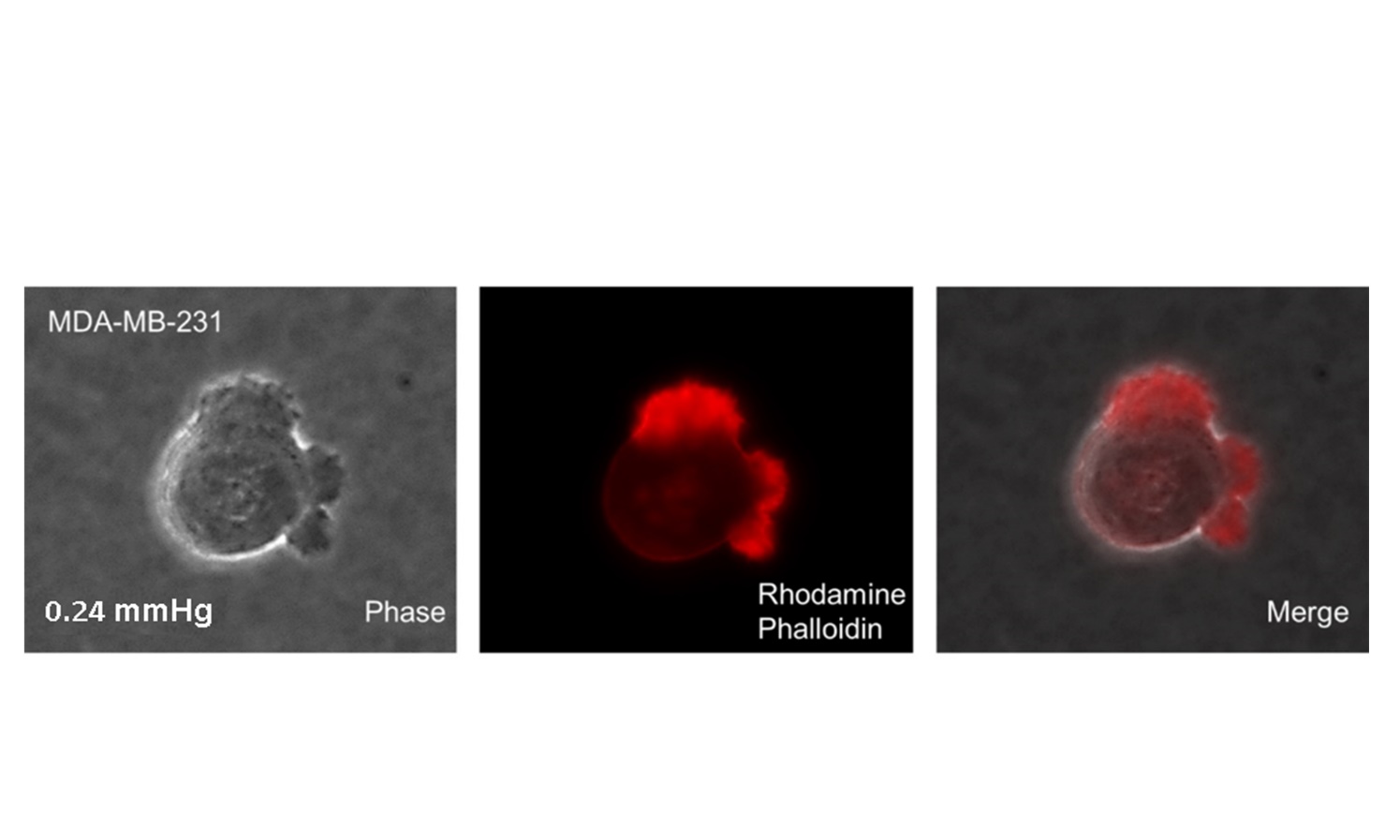


**Supplementary Figure 2.** Representative images of Rhodamine Phalloidin stained MDA-MB-231 cells under 0.24 mmHg compression force.


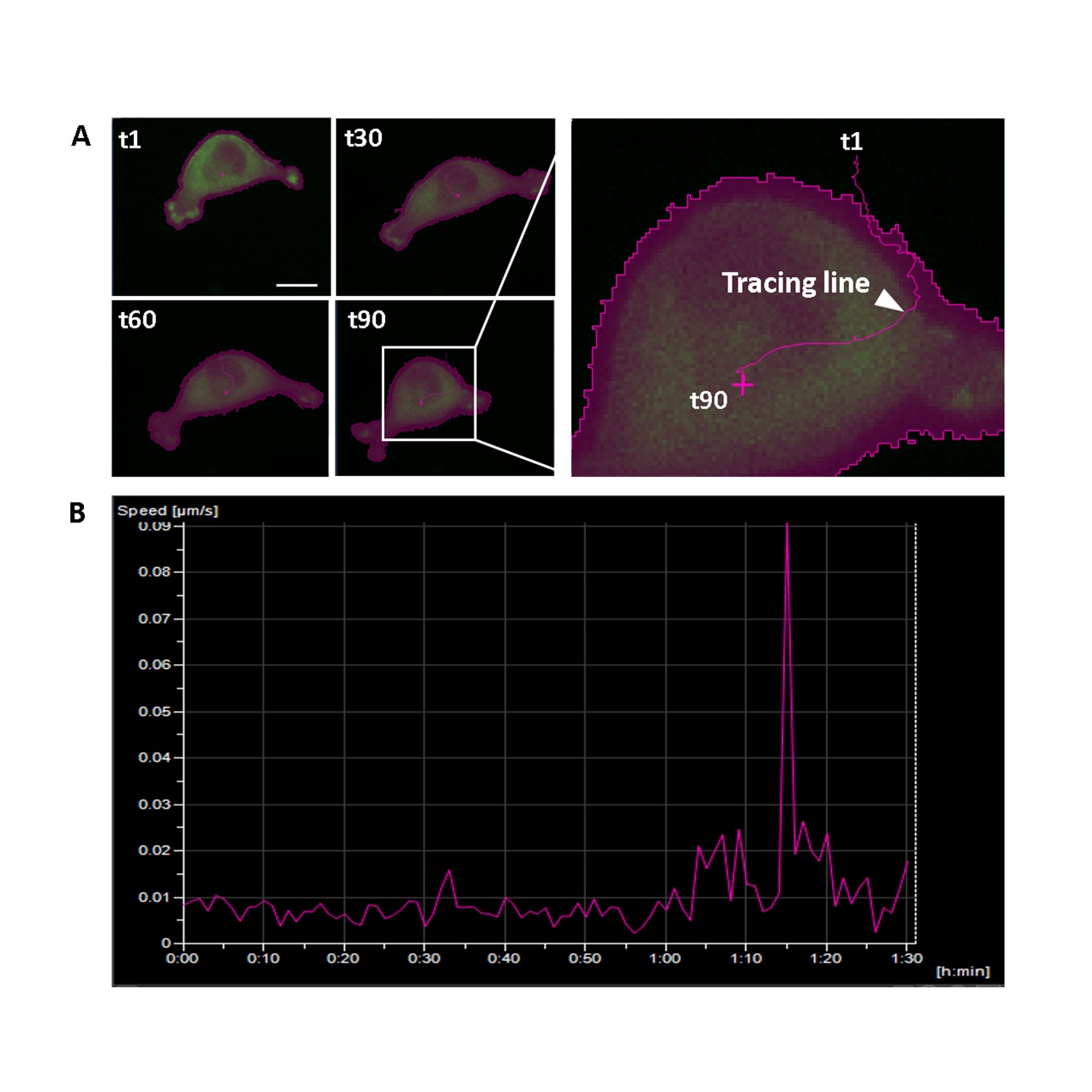


**Supplementary Figure 3.** Trajectory and migration speed of a migrating MDA-MB-231cancer cell under compression on a low adhesive-surface condition. (A) Images showing the cell's morphology and location change over time. (B) Analyzed cell migration speed from time-lapse sequential images. The x-axis is time; the y-axis is motile speed. Scale bar = 20 μm.


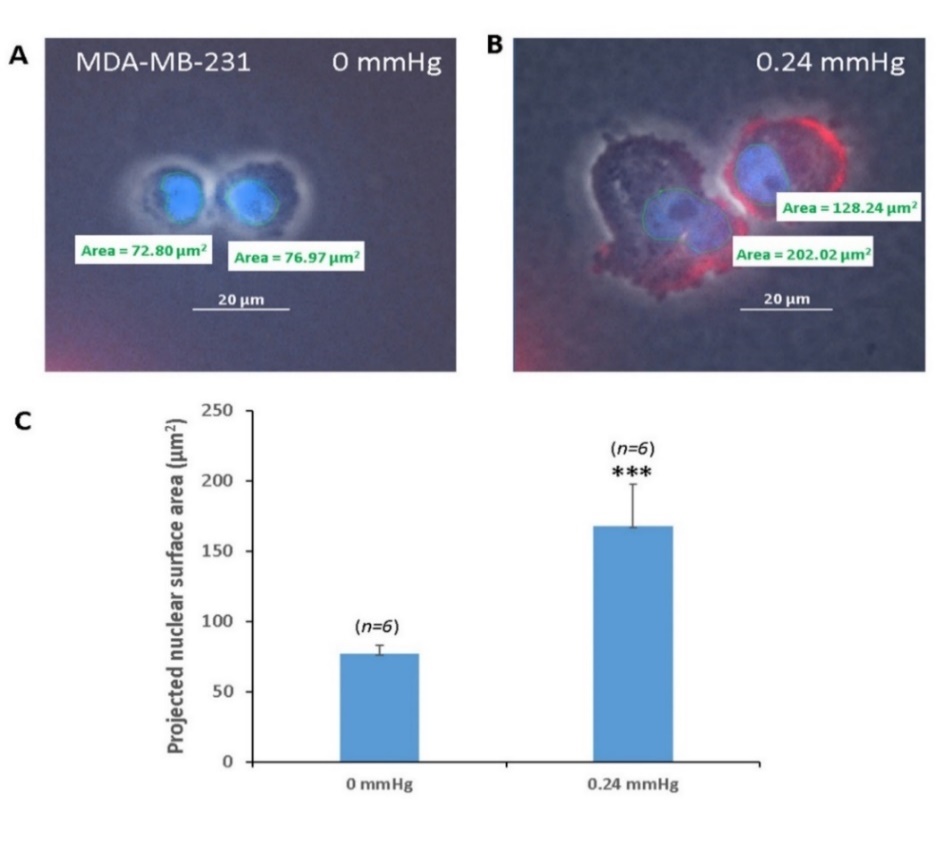


**Supplementary Figure 4.** Compression force altered cell nuclear area of MDA-MB-231 cancer cells on a low-adhesive surface. Representative images of the cell nucleus of MDAMB231 without (A) and with (B) compressive force. (C)The cells' projected nucleus surface areas are without and with compressive force. Scale bar = 20 μm. ***p < 0.005. Student’s t-test, two independent experiments. Rhodamine phalloidin (Red), Hoechst 33342 (Blue).


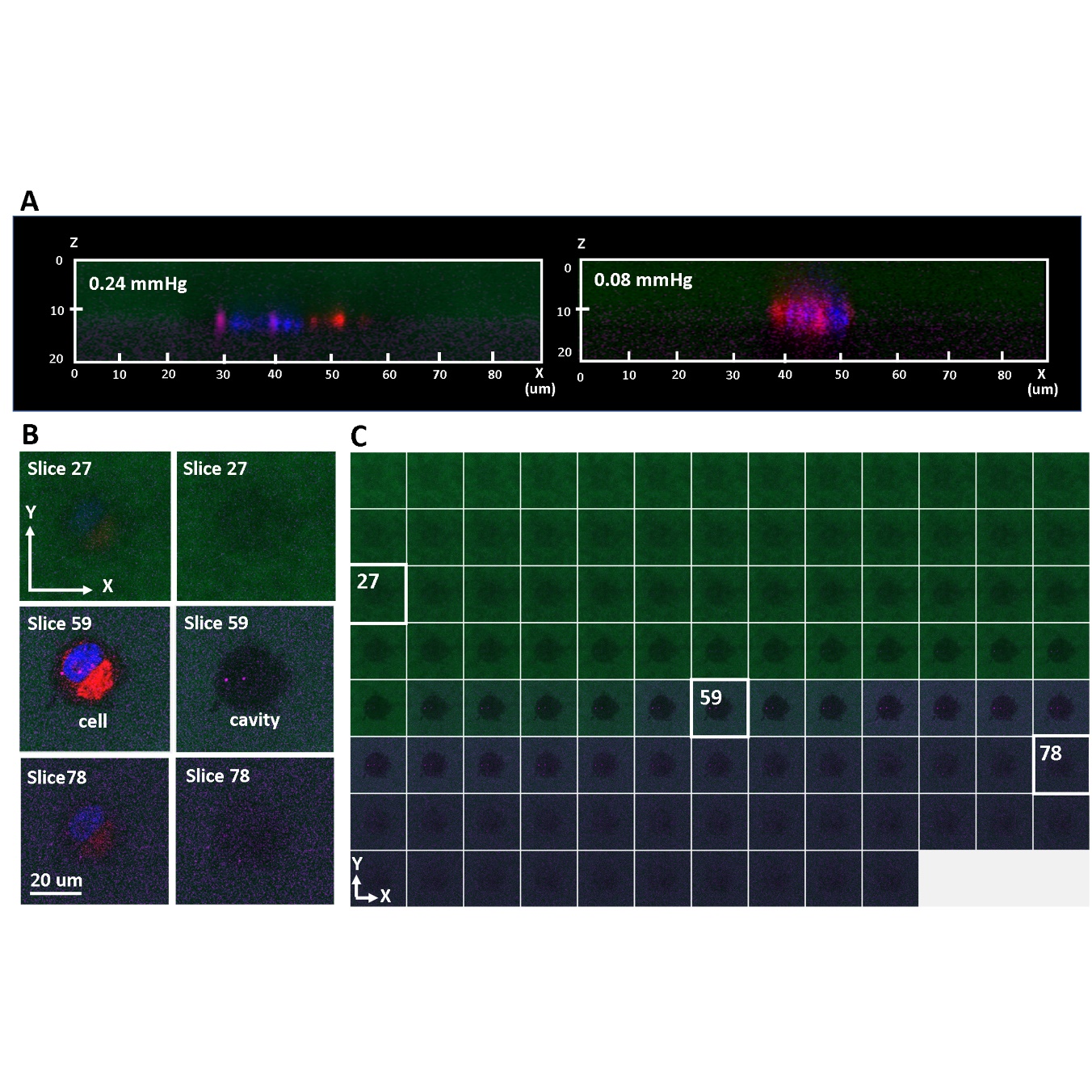


**Supplementary Figure 5.** Confocal images of a sandwiched cell at the gel-gel interface. (A) 3D rendered confocal images (viewed from the side) showing a single MDA-MB-231 cell sandwiched at the gel-gel interface under 0.24 mmHg (left) and 0.08 mmHg (right) compressive stress. (B) Three Z-section confocal images obtained from a single MDA-MB-231 cell under 0.24 mmHg compressive stress at the gel-gel interface (left column), and the same images without showing the fluoresce channels (DAPI and TRITC) of the cell (right column). (C) The entire Z-section confocal images of the single MDA-MB-231 cell under 0.24 mmHg compressive stress at the gel-gel interface without showing the fluoresce channels (DAPI and TRITC). The gel on the top (shown in green) was pre-labeled with Dextran-FITC whereas the gel at the bottom was pre-labeled with antigoat-cy5. The cell's nucleus and lipids was stained with Hoechst 33342 and DilC12, respectively.

Supplementary Movies

**Video S1 Description:** Compression force induces transition to lamellipodia protrusions on a low-adhesive surface. MDA-MB-231 cells were transfected with actin-GFP using a baculovirus vector and then seeded onto a low-adhesive agarose gel surface on our device. Then, agarose gel was placed on top of the cells to deliver a compression force of 0.24 mmHg. The cells were allowed to sediment for 60 minutes in the incubator before movie capture by a time-lapse microscope. The spread out morphology and actin filament assembly at the tip of the cell protrusions are typical of the lamellipodia protrusion phenotype in mesenchymal migrating cells. GFP, green fluorescent protein.

**Video S2 Description:** Cell motility via blebby protrusions on a low-adhesive agarose gel surface. MDA-MB-231 cells were transfected with actin-GFP using a baculovirus vector and then seeded onto a low-adhesive agarose gel surface on our device. The cells were allowed to sediment for 60 minutes in an incubator before movie capture by a time-lapse microscope. The round morphology and rapid movements of the cell protrusions are typical of the blebby protrusion phenotype in amoeboid migrating cells. GFP, green fluorescent protein.

**Video S3 Description:** Cell motility is autonomous on a low-adhesive agarose gel surface. MDA-MB-231 cells were co-seeded with reference polystyrene microbeads whose size and mass density were close to those of the cells (1.05 g/cm^3^, 10 μm diameters) onto the low-adhesive agarose gel surface on our device. The microbeads remained mostly stationary during the experiment, indicating that the movement of cells is autonomous.

**Video S4 Description:** Trajectory and migration speed of a migrating MDA-MB-231cancer cell under 0.24 mmHg compressive stress condition on a low adhesive surface during a 92 minutes time course. The cells moved in different directions indicates that the cells' movements were autonomous instead of due to lateral drift of the stacking gels.
